# Lower Geriatric Nutritional Risk Index is associated with increased risk of acromial stress fracture following reverse total shoulder arthroplasty

**DOI:** 10.1016/j.jsea.2026.100085

**Published:** 2026-08-20

**Authors:** Leonard Stokes, Vidhur Polam, Yousy Martinez Sauvanell, Tammy Hoffman, Michelle Gosselin, Andrew Nahr, Patrick J. Denard

**Affiliations:** a Oregon Shoulder Institute, Medford, OR, USA; b Ponce Health Science University, Ponce, Puerto Rico; c Oregon Health & Science University, Portland, OR, USA

**Keywords:** Reverse shoulder arthroplasty, GNRI, Nutrition, Stress fracture, Scapular spine, Acromion, Complication risk

## Abstract

**Background:**

Acromial and scapular spine stress fractures are serious complications following reverse total shoulder arthroplasty (rTSA), and patient-level risk factors may contribute to their development. The Geriatric Nutritional Risk Index (GNRI) is a validated marker of physiologic reserve. The purpose of this study was to evaluate the association between GNRI and post-operative stress fracture after rTSA. We hypothesized that lower GNRI would be associated with increased fracture risk.

**Methods:**

We performed a retrospective 1:2 age- and sex-matched case-control study of patients undergoing primary rTSA. Forty-five patients who developed post-operative acromial or scapular spine stress fracture were matched to 90 controls without fracture. Demographic variables collected included age, sex, body mass index, diagnosis, and comorbidities. GNRI was calculated pre-operatively and post-operatively and analyzed as a continuous variable and using predefined thresholds (<98, <103, <109). Conditional logistic regression adjusted for diagnosis and comorbidities. Receiver-operating characteristic analysis assessed discriminatory ability.

**Results:**

A total of 135 patients were included (mean age, 72.8 years; 53% female). Patients with stress fracture had lower pre-operative GNRI (107.4 ± 12.4 vs. 114.0 ± 13.9; *P* = .007) and post-operative GNRI (105.5 ± 14.9 vs. 114.8 ± 14.5; *P* = .001). Post-operative GNRI <109 was more common among fracture patients (60% vs. 30%; *P* = .001). In matched multivariable analysis, each 1-point increase in post-operative GNRI was associated with a 5% reduction in fracture odds (odds ratioOR, 0.95; 95% confidence interval [CI], 0.92-0.99; *P* = .015). Post-operative GNRI <109 was independently associated with fracture (odds ratio, 3.64; 95% CI, 1.02-13.02; *P* = .047). Receiver-operating characteristic analysis demonstrated moderate discrimination (area under the curve, 0.70; 95% CI, 0.60-0.79).

**Conclusion:**

In this matched case-control analysis, lower post-operative GNRI was independently associated with increased risk of acromial and scapular spine stress fracture following rTSA. GNRI may serve as a practical perioperative risk stratification tool in patients undergoing reverse shoulder arthroplasty.

Acromial and scapular spine stress fractures are a challenging complication following reverse total shoulder arthroplasty (rTSA), associated with increased pain, functional limitations, and inferior outcomes.^5^^,^^8^ Reported incidence ranges from <1% to >10%, varying with patient characteristics and imaging sensitivity.^13^^,^^15^^,^^28^^,^^31^^,^^35^ More recent cohorts incorporating advanced or routine imaging have estimated the true incidence to be approximately 7%-8%.^13^^,^^14^

Several implant-related risk factors have been proposed to contribute to fracture risk, including inferior glenosphere positioning and onlay humeral stems, which have the potential to increase deltoid tension and scapular strain.^10^^,^^13^^,^^27^^,^^23^ Conversely, other studies have suggested that specific implant configurations may mitigate acromial stress, highlighting the complex relationship between implant design and fracture risk.^23^^,^^27^ However, the influence of implant design features remains controversial, as some studies demonstrate associations while others report no consistent relationship.^10^^,^^13^^,^^23^ Additionally, systemic and host-level factors are known contributors to fracture susceptibility. Noble et al^26^ suggested that host-level characteristics are more significant than pre-operative radiographic characteristics in predicting fracture risk. The American Shoulder and Elbow Surgeons (ASES) Complications Research Group also emphasized the additive role of systemic health and implant configuration in fracture development.^1^

Several host factors have been shown to contribute to fracture risk, including osteoporosis, renal dysfunction, and low body mass index (BMI).^2^^,^^24^ Patients with autoimmune diseases such as rheumatoid arthritis may be particularly vulnerable because of chronic inflammation and compromised bone quality.^3^ Despite the relationship between bone density and nutritional status, which can serve as an upstream determinant of both bone integrity and healing capacity, few studies have examined the association between post-operative stress fracture after rTSA and this potentially modifiable factor.

Optimization of perioperative nutritional status has been recognized as a critical component of enhanced recovery and complication prevention.^32^^,^^21^ The Geriatric Nutritional Risk Index (GNRI), developed by Bouillanne et al,^4^ is a validated index based on serum albumin and body weight that quantifies nutritional risk. GNRI has demonstrated prognostic value across multiple orthopedic populations, including patients undergoing hip fracture repair, spine surgery, and lower extremity arthroplasty.^9^^,^^11^^,^^12^^,^^17^^,^^33^ Low GNRI independently predicts post-operative complications and mortality.^6^^,^^19^^,^^20^^,^^25^^,^^34^ With regard to shoulder arthroplasty, low GNRI has also been linked to increased risk of complications including infection, delayed wound healing, prolonged hospitalization, and higher readmission rate.^18^

The primary purpose of the current study was to evaluate the relationship between GNRI and risk of acromial stress fracture following rTSA. We further examined whether post-operative GNRI, reflecting early perioperative physiologic reserve, was more predictive than pre-operative values. We hypothesized that GNRI would be associated with increased risk of post-operative acromial and scapular spine stress fractures following rTSA.

## Materials and methods

### Study design

This was a retrospective, single-center, 1:2 age- and sex-matched case-control study conducted following institutional review board approval. A database of patients who underwent primary rTSA between 2011 and 2024 was reviewed to identify patients with post-operative acromial or scapular spine stress fracture. Inclusion criteria were primary rTSA, a minimum 1-year clinical follow-up, and complete GNRI and demographic data. Exclusion criteria included pre-operative acromial or scapular spine fracture, revision arthroplasty, rTSA for tumor or fracture, previous shoulder fracture, or missing nutritional data.

Forty-five patients were identified that developed a post-operative acromial or scapular spine stress fracture during the study period were matched to 90 control patients without post-operative fracture by exact sex and nearest age without replacement. Matching prioritized controls within ±2 years of age, with expansion of the age caliper when necessary. The maximum observed age difference was 4 years. All rTSAs were performed by a single surgeon using a deltopectoral approach, a 135° press-fit humeral component designed for inlay position (Univers Revers; Arthrex Inc.; Naples, FL) and a lateralized glenoid component (Universal Baseplate or Modular Glenoid System; Arthrex, Inc.), consistent with the senior surgeon's standard surgical technique.

Demographic characteristics and comorbidities (eg tobacco use, renal disease, diabetes, and osteoporosis) were collected from the electronic medical record. Race and ethnicity data were not available in the registry dataset. BMI and GNRI values were calculated for both groups. Formal bone mineral density measurements (eg, dual-energy X-ray absorptiometry) were not routinely available for all patients. Osteoporosis medication use documented in the medical record was, therefore, used as the available surrogate measure of bone health.

### Geriatric Nutritional Risk Index calculation

Serum albumin, height, and weight available from routine perioperative clinical care were retrospectively extracted from the electronic medical record to calculate the GNRI. The GNRI was calculated using formula 1, and ideal body weight was calculated with formula 2 measuring height in meters.

1.GNRI=(1.489×serumalbumin[g/L])+(41.7×presentbodyweight[kg]/idealbodyweight[kg])

$$2.\;Ideal\;Body\;Weight=\left(22\times {height}^{2}\right)$$

GNRI was calculated at 2 time points: pre-operatively (within 90 days before surgery) and post-operatively (between 4 and 12 weeks after surgery). GNRI was analyzed as both a continuous variable and dichotomized using predefined thresholds of <98, <103, and <109 based on prior orthopedic literature.^4^^,^^18^^,^^30^

### Outcomes

The primary outcome was the occurrence of a post-operative stress fracture involving the acromion or scapular spine. The diagnosis was based on post-operative radiographs and/or computed tomography.^22^^,^^29^ Fractures were categorized using the Levy classification: type I (acromial tip), type II (acromial base), or type III (scapular spine).^16^ Time to fracture diagnosis was recorded in weeks from the date of surgery.

### Statistical analysis

Continuous variables were reported as means ± standard deviation and compared using independent or paired *t*-tests, as appropriate. Categorical variables were compared using Fisher's exact or chi-square tests. Among fracture patients, time to diagnosis across Levy classification types was evaluated using one-way analysis of variance (ANOVA) with confirmation by Kruskal–Wallis testing.

Given the 1:2 age- and sex-matched design, conditional logistic regression was used as the primary analytic method to evaluate the association between GNRI and post-operative stress fracture while accounting for matched strata. Although GNRI was calculated at both pre-operative and post-operative time points, post-operative GNRI was selected for the primary multivariable and receiver-operating characteristic (ROC) analyses because it reflects both baseline nutritional status and the early physiologic response to surgery, thereby representing perioperative physiologic reserve. Pre-operative GNRI was evaluated descriptively and in univariable analyses. Multivariable models adjusted for diagnosis (cuff tear arthropathy [CTA] vs. osteoarthritis), BMI, tobacco use, renal disease, endocrine disorder, inflammatory arthritis, and osteoporosis medication use. Six patients were excluded from multivariable analyses because of incomplete covariate data, leaving 129 patients across 44 matched sets for conditional logistic regression. Because cases and controls were matched by sex, sex-stratified analyses were not performed. Post-operative GNRI was analyzed as both a continuous variable (per 1-point increase) and as dichotomized thresholds (<98, <103, <109),^4^^,^^18^^,^^30^ with continuous and threshold models evaluated separately. Sensitivity analyses excluding inflammatory arthritis alone and inflammatory arthritis plus tobacco use were performed to assess robustness. ROC analysis evaluated the discriminatory ability of continuous post-operative GNRI, with the optimal cutoff determined using the Youden index. Statistical significance was defined as *P* < .05.

## Results

### Baseline characteristics

Baseline characteristics of the cohorts with and without stress fracture are shown in Table I. BMI was lower in the fracture group (26.5 ± 5.4 vs. 29.7 ± 6.9; *P* = .004). CTA (78% vs. 52%; *P* = .005) and inflammatory arthritis were more common among patients with fracture (24% vs. 6%; *P* = .003). Tobacco use (13% vs. 38%; *P* = .005) and renal disease (9% vs. 29%; *P* = .008) were more common among controls.

**Table I:** Baseline demographics, comorbidities, and nutritional characteristics between stress fracture and matched control cohorts.

Variable	Stress fracture group (n = 45)	Control group (n = 90)	*P* value
Demographics			
Age (yr)	72.8 ± 7.2	72.8 ± 6.1	.958
Female sex	25 (56%)	46 (51%)	.715
BMI (kg/m^2^)	26.5 ± 5.4	29.7 ± 6.9	.004
Diagnosis			
Primary osteoarthritis	10 (22%)	43 (48%)	.005
Cuff arthropathy	35 (78%)	47 (52%)	.005
Comorbidities			
Tobacco use	6 (13%)	34 (38%)	.005
Renal disease	4 (9%)	26 (29%)	.008
Endocrine disorder	15 (33%)	37 (41%)	.454
Inflammatory arthritis	11 (24%)	5 (6%)	.003
Osteoporosis	6 (13%)	5 (6%)	.179
GNRI			
Pre-operative GNRI	107.4 ± 12.4	114.0 ± 13.9	.007
Post-operative GNRI	105.5 ± 14.9	114.8 ± 14.5	.001
ΔGNRI (post–pre)	−1.9 ± 4.1	+0.8 ± 3.6	.352
GNRI thresholds			
Pre-operative			
<98	5 (11.1%)	7 (7.1%)	.518
<103	14 (31.1%)	17 (17.3%)	.064
<109	26 (57.8%)	34 (34.7%)	.009
Post-operative			
<98	10 (22.2%)	6 (6.1%)	.005
<103	17 (37.8%)	16 (16.3%)	.005
<109	27 (60.0%)	29 (29.6%)	.001

*BMI*, body mass index; *GNRI*, Geriatric Nutritional Risk Index; *n*, number of patients; *ΔGNRI*, post-operative minus preoperative.

Pre-operative GNRI was lower in the fracture group (107.4 ± 12.4) than in controls (114.0 ± 13.9; *P* = .007). A greater proportion of fracture patients had GNRI <109 pre-operatively (58% vs. 30%; *P* = .009), whereas pre-operative thresholds of <98 (11% vs. 7%; *P* = .518) and <103 (31% vs. 17%; *P* = .064) were not significantly different between groups.

Post-operative GNRI was also lower in the fracture group (105.5 ± 14.9 vs. 114.8 ± 14.5; *P* = .001). Post-operatively, GNRI <98 (22% vs. 6%; *P* = .005), <103 (38% vs. 16%; *P* = .005), and <109 (60% vs. 30%; *P* = .001) were all more common among fracture patients. Among patients with pre-operative GNRI ≥109, 62% of the fracture group declined below 109 post-operatively versus 3% of controls (*P* < .001).

#### Fracture characteristics

Among patients who developed stress fractures (n = 45), the mean time to diagnosis was 5.0 ± 5.4 months following surgery. Distribution by Levy classification was type 1 (26.7%), type 2 (53.3%), and type 3 (20.0%). Time to fracture did not differ significantly by Levy classification (ANOVA *P* = .660; Kruskal–Wallis *P* = .230). Pre-operative and post-operative GNRI values did not significantly differ across Levy fracture types.

#### Regression and sensitivity analysis

In matched conditional logistic regression adjusting for diagnosis and comorbidities, lower post-operative GNRI was independently associated with stress fracture risk (odds ratio [OR], 0.95 per 1-point increase; 95% confidence interval [CI], 0.92-0.99; *P* = .015) (Table II). CTA diagnosis (OR, 3.01; 95% CI, 1.06-8.56), inflammatory arthritis (OR, 6.95; 95% CI, 1.44-33.55), and current tobacco use (OR: 25.26; 95% CI: 1.78-358.39) were associated with fracture, whereas renal disease demonstrated an inverse association (OR, 0.08; 95% CI, 0.01-0.79).

**Table II:** Levy classification and associated pre-operative and post-operative GNRI values in patients with stress fracture.

Levy classification	n (%)	Pre-operative GNRI (median [IQR])	Post-operative GNRI (median [IQR])
Type 1	12 (26.7%)	108.6 (104.4-110.1)	106.9 (98.0-115.9)
Type 2	24 (53.3%)	106.4 (101.7-111.4)	106.8 (101.7-111.9)
Type 3	9 (20.0%)	110.2 (103.3-117.1)	109.3 (96.2-112.2)

*GNRI*, Geriatric Nutritional Risk Index; *IQR*, interquartile range; *n*, number of patients.

In threshold models, post-operative GNRI <109 (OR, 3.64; 95% CI, 1.02-13.02; *P* = .047), <103 (OR, 9.25; 95% CI, 1.57-54.51; *P* = .014), and <98 (OR, 7.05; 95% CI, 1.35-36.95; *P* = .021) were associated with increased fracture risk (Table III).

**Table III:** Conditional logistic regression analysis of factors associated with post-operative stress fracture risk.

Predictor	OR	95% CI	*P* value
Post-operative GNRI continuous model			
Post-operative GNRI (per 1-point increase)	0.95	0.92-0.99	.015
CTA diagnosis (vs. OA)	3.01	1.06-8.56	.039
BMI (kg/m^2^)	0.99	0.90-1.08	.771
Current tobacco use	25.26	1.78-358.39	.017
Renal disease	0.08	0.01-0.79	.03
Endocrine disorder	0.75	0.26-2.16	.597
Inflammatory arthritis	6.95	1.44-33.55	.016
Osteoporosis medication use	1.21	0.26-5.73	.81
GNRI threshold models			
<109	3.64	1.02-13.02	.047
<103	9.25	1.57-54.51	.014
<98	7.05	1.35-36.95	.021

*BMI*, body mass index; *CI*, confidence interval; *CTA*, cuff tear arthropathy; *GNRI*, Geriatric Nutritional Risk Index; *OA*, osteoarthritis; *OR*, odds ratio.

Sensitivity analyses excluding inflammatory arthritis and inflammatory arthritis plus tobacco use demonstrated consistent directionality and maintained statistical significance for continuous post-operative GNRI (Table IV).

**Table IV:** Sensitivity analyses excluding high-risk comorbidity subgroups (CTA-adjusted).

Excluded comorbidity	OR	CI	*P* value
Inflammatory arthritis			
Continuous	0.96	0.92-0.99	.025
<98	4.99	1.00-24.85	.049
<103	8.51	1.13-64.36	.038
<109	3.46	0.81-14.70	.093
Inflammatory arthritis and tobacco			
Continuous	0.95	0.91-0.99	.023
<98	3.94	0.82-19.04	.088
<103	9.13	1.06-78.39	.044
<109	3.55	0.79-15.91	.099
Inflammatory arthritis and renal disease			
Continuous	0.96	0.92-1.00	.032
<98	4.53	0.91-22.58	.066
<103	6.06	0.88-41.59	.067
<109	3.34	0.66-16.87	.144

*CI*, confidence interval; *CTA*, cuff tear arthropathy; *OR*, odds ratio.

#### Receiver-operating characteristic analysis

**Figure 1:**
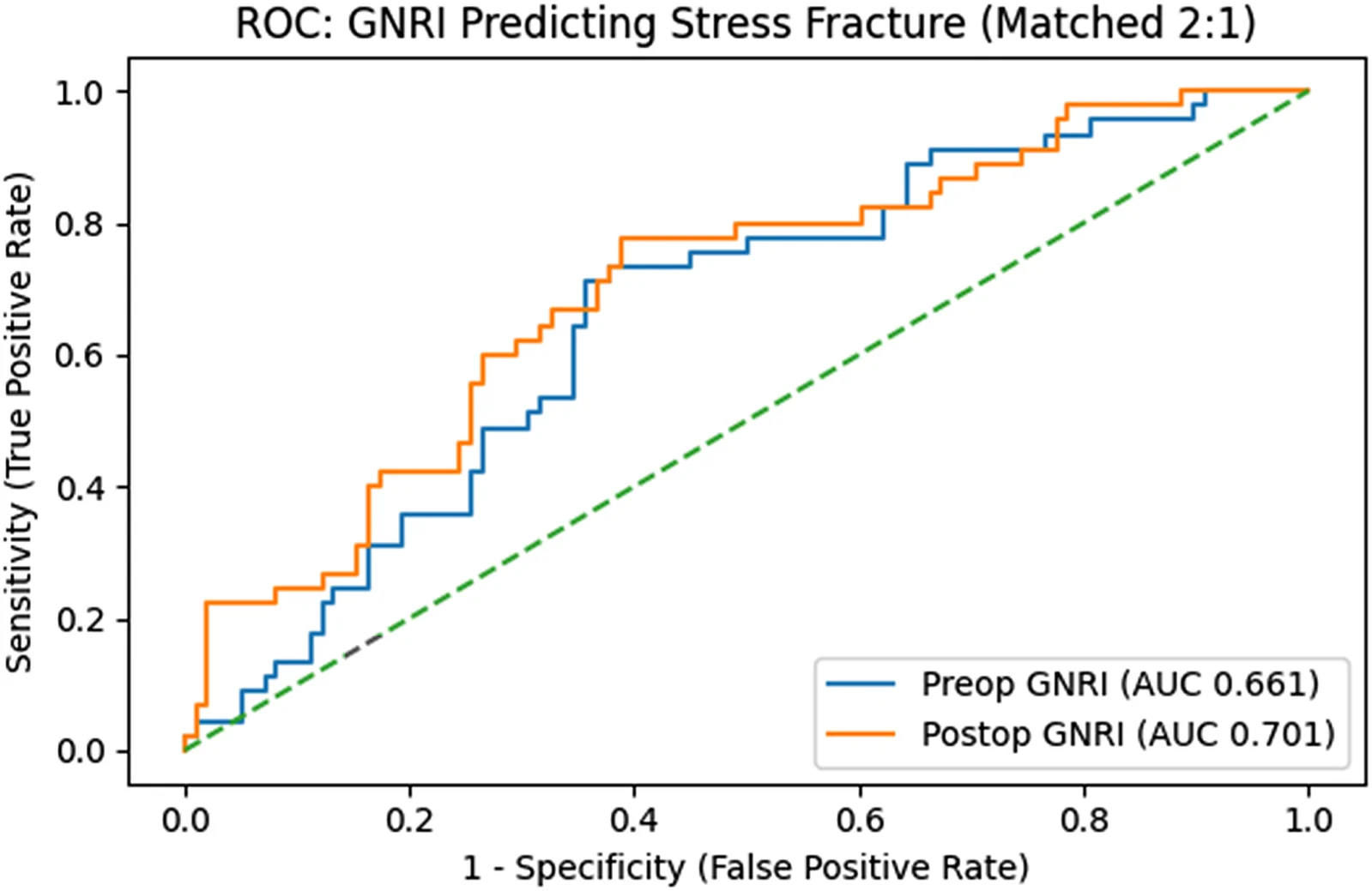
ROC analysis for prediction of post-operative acromial or scapular spine stress fracture using pre-operative and post-operative GNRI values. Post-operative GNRI demonstrated greater predictive discrimination for stress fracture risk compared with pre-operative GNRI (AUC 0.701 vs. 0.661) in the matched 2:1 cohort. *AUC*, area under the curve; *GNRI*, Geriatric Nutritional Risk Index; *ROC*, receiver-operating characteristic.

ROC analysis of continuous post-operative GNRI demonstrated moderate discrimination (area under the curve [AUC], 0.701; 95% CI, 0.604-0.794) (Fig. 1). The optimal Youden-derived cutoff was approximately 113, yielding a sensitivity of 77.8% and specificity of 61.2%.

## Discussion

In this matched case-control analysis, lower post-operative GNRI was associated with post-operative acromial and scapular spine stress fractures following rTSA. In conditional logistic regression accounting for matched strata and comorbidities, each 1-point increase in post-operative GNRI was associated with a 5% reduction in fracture odds (OR: 0.95; 95% CI, 0.92-0.99). Threshold analyses demonstrated increased fracture risk at <109, <103, and <98, and ROC analysis demonstrated moderate discrimination (AUC, 0.701). Associations persisted across sensitivity analyses excluding patients with inflammatory arthritis and both inflammatory arthritis and current tobacco use, suggesting that the observed association between post-operative GNRI and stress fracture was not driven solely by these high-risk subgroups.

Symptomatic acromial and scapular spine stress fractures occur in approximately 3%-4% of patients following rTSA, most commonly within the first post-operative year.^2^ In the ASES Complications of rTSA Multicenter Study of 6,755 shoulders, the overall incidence was 3.9%, with fractures occurring at a mean of 8.2 months post-operatively.^2^ Independent patient-level predictors included inflammatory arthritis (OR, ∼2.1-2.2), female sex (OR: 1.5-2.3), osteoporosis (OR, ∼2.2-2.6), and rotator cuff arthropathy.^2^^,^^24^ Other multicenter work demonstrated that fracture incidence varies by indication, with higher rates in CTA and massive cuff tear cohorts (5.2%) compared with glenohumeral osteoarthritis (2.1%), although inflammatory arthritis remained predictive across diagnostic strata (OR, 1.9-2.9).^1^ Noble et al^26^ reported that after adjustment and correction for multiple comparisons, inflammatory arthritis in females conferred nearly fivefold increased odds of fracture (OR, 4.87), while several radiographic parameters lost statistical significance, suggesting that patient characteristics may outweigh pre-operative imaging findings in fracture risk stratification. In our age- and sex-matched case-control study, inflammatory arthritis remained independently associated with stress fracture (OR, 6.95), whereas female sex was not, further emphasizing the primacy of patient-level vulnerability over demographic or radiographic factors alone in determining fracture risk following rTSA.

The GNRI was developed as a composite of serum albumin and ideal body weight to stratify morbidity and mortality risk in elderly hospitalized patients.^4^ Importantly, GNRI functions as a graded risk prediction tool, with progressively lower values associated with stepwise increases in complications and mortality rather than serving as a binary diagnosis of malnutrition.^4^ In shoulder arthroplasty populations, moderate and severe GNRI-defined malnutrition have been independently associated with increased post-operative complications, readmission, prolonged hospitalization, and mortality, demonstrating a dose-dependent relationship between nutritional risk and surgical outcomes.^18^ Beyond general perioperative risk, low GNRI has been associated with osteoporosis (OR, 1.33)^19^ and with impaired tendon healing after rotator cuff repair, where GNRI <103 independently predicted structural failure at 2 years (OR, 3.88-5.62).^30^ In the present matched case-control study, post-operative GNRI demonstrated a similar graded association with stress fracture risk, with each 1-point increase corresponding to a 5% reduction in fracture odds (OR: 0.95) and a consistent effect direction maintained across sensitivity analyses. These findings suggest that GNRI may reflect systemic physiologic reserve—including bone quality and tissue resilience—rather than isolated nutritional deficiency, providing biologic plausibility for its association with stress fracture following rTSA.

In a large national cohort, moderate malnutrition (GNRI, 92-98) was independently associated with increased complications (OR, 1.74), while severe malnutrition (<92) conferred more than threefold higher odds of adverse events and over fourfold increased mortality.^18^ In the present study, post-operative GNRI demonstrated moderate discrimination for stress fracture risk (AUC, 0.701), with continuous modeling revealing a dose-dependent association rather than reliance on a single threshold. These findings suggest that GNRI may serve as a practical risk stratification tool within rTSA populations. Current Enhanced Recovery After Surgery guidelines and perioperative consensus statements strongly recommend routine pre-operative nutrition screening and optimization for surgical patients, recognizing suboptimal nutritional status as an independent predictor of poor outcomes.^21^^,^^32^ Although it remains unknown whether targeted nutritional intervention reduces stress fracture risk, emerging prehabilitation data demonstrate that perioperative nutritional risk can be improved through structured multimodal programs.^7^ Together, these findings support incorporation of GNRI into perioperative risk assessment while underscoring the need for prospective investigation before therapeutic recommendations can be made.

### Limitations

This study has limitations inherent to its retrospective design, including potential residual confounding despite age- and sex-matched conditional logistic regression with multivariable adjustment. GNRI was derived from routine laboratory data and may be influenced by systemic inflammation because of its incorporation of serum albumin. Additionally, micronutrient deficiencies were not routinely assessed; therefore, GNRI should be interpreted as a marker of overall physiologic reserve rather than isolated nutritional deficiency. The fracture cohort was modest (n = 45), resulting in wide confidence intervals for several regression estimates, particularly the threshold analyses. Additionally, renal disease was recorded as a binary variable without information regarding chronic kidney disease stage or severity, and tobacco exposure was categorized as never, former, or current use without quantification of cumulative exposure. Consequently, these secondary associations should be interpreted cautiously, as residual confounding cannot be excluded. Complete bone mineral density data and micronutrient assessments were not available for all patients, and nutritional supplementation data were lacking. Race and ethnicity data were not available, limiting assessment of potential disparities and affecting generalizability. Additionally, although laboratory intervals were standardized, not all values were obtained concurrently. Finally, this was a single-center study utilizing a single implant design; evaluated GNRI thresholds were based on orthopedic literature and cohort-specific discrimination rather than traditional mortality categories, which may limit generalizability. These findings warrant prospective validation across multiple centers.

## Conclusion

In this matched case-control analysis, lower postoperative GNRI was independently associated with increased risk of acromial and scapular spine stress fracture following rTSA. GNRI may serve as a practical perioperative risk stratification tool in patients undergoing reverse shoulder arthroplasty.

## Disclaimers:

Funding: No funding was disclosed by the authors.

Conflicts of interest: Patrick J. Denard has received consulting fees and royalties from Arthrex for work unrelated to the subject of this article. Any additional authors, their immediate families, and any research foundations with which they are affiliated have not received any financial payments or other benefits from any commercial entity related to the subject of this article.
